# A qualitative study of lived experiences and needs of advanced cancer patients in Malaysia: Gaps and steps forward

**DOI:** 10.1007/s11136-023-03401-5

**Published:** 2023-03-24

**Authors:** Alene Sze Jing Yong, Mark Wing Loong Cheong, Ednin Hamzah, Siew Li Teoh

**Affiliations:** 1 School of Pharmacy, Monash University Malaysia, Subang Jaya, Selangor Malaysia; 2 Hospis Malaysia, Kuala Lumpur, Malaysia

**Keywords:** Malignancy, Quality of life, Developing country, Health services needs, Barriers

## Abstract

**Purpose:**

Due to the high burden of cancer-related suffering, it is paramount to understand the gaps in cancer care that lead to suffering. Advanced cancer patients have unmet needs and challenges that differ from those with early-stage cancer due to the rapid disease progression. By exploring advanced cancer patients' lived experiences and needs from the physical, psychological, social, and spiritual aspects, this study aims to identify gaps in the Malaysian health system and propose contextualised measures to improve cancer care.

**Methods:**

Semi-structured, in-depth interviews were conducted to explore advanced cancer patients' lived experiences and needs from the physical, psychological, social, and spiritual aspects. The interviews were then transcribed and coded. Themes were developed from the codes using iterative thematic approach.

**Results:**

The lived experiences and needs of nineteen patients converged into four major themes: disruption to daily lives, psychosocial and spiritual support system, information needs, and financial needs. This study described predominantly how cancer impacted patients’ lives and livelihood, how patients coped with their psychological conditions after diagnosis, the need for effective communication and trust in a multicultural society, and how finance affected access to and experience of cancer care.

**Conclusion:**

Advanced cancer patients had different needs beyond receiving medical treatments. A concerted effort is required from clinicians, allied health professionals, social workers, support groups, and family members to understand and fulfil these needs.

## Plain English summary

Advanced cancer patients often endure high degrees of suffering. It is paramount to understand the gaps in cancer care to minimise suffering. This study aims to have an in-depth understanding of advanced cancer patients' unique experiences within the first year after being diagnosed with cancer. We explored how their quality of life can be improved from the physical, psychological, social, and spiritual aspects. Lastly, we proposed measures to improve cancer care based on the gaps we found. From the interview with nineteen patients, we described patients' various needs beyond medical treatments. We described how cancer impacted patients' lives because they could no longer carry out their daily activities or fulfil family responsibilities. They often coped with their emotional needs through religion, friends, and family support. The impact of cancer on livelihood is particularly prominent in the lower-income group due to the lack of employment protection. A holistic health system should provide financial support to cancer patients in terms of income replacement protection schemes and other non-medical costs while accessing care. Clinicians must consider cultural contexts while providing health information to build trust between clinicians and patients. A concerted effort is required from clinicians, allied health professionals, social workers, support groups, and family members to understand and fulfil advanced cancer patients' needs.

## Introduction

Cancer is the second leading cause of death and disability due to non-communicable diseases worldwide, contributing to 10 million deaths in 2019 [1]. Despite the improved survival rate due to advancements in cancer therapy, it was estimated that 90% of deceased cancer patients endured tremendous suffering, compromising their physical, social, spiritual, and emotional functionings that could not be relieved without medical intervention [2, 3].

Due to the high burden of cancer-related suffering, it is paramount to understand the gaps in cancer care that lead to suffering. As much as life extension is vital in cancer care, patients value their quality of life (QoL) equally [4]. Previous literature has established that cancer patients with more unmet needs are associated with lower QoL [5–7]. The most commonly reported unmet needs are psychosocial (10.1–84.4%), information (4–66.7%), and physical needs (17–48%) [8–10]. Despite the abundance of literature about patients' needs, evidence from low-and-middle-income countries (LMICs) remains scarce [11]. Findings generated from high-income countries (HICs) might lack generalisability in LMICs due to the differences in health systems, cultures, and resources. LMICs are postulated to be disproportionately burdened by the serious suffering from cancer, estimated to increase by 109% between 2016 and 2060 [2]. Previous study has also reported that LMICs have higher unmet needs than HICs in accessing comprehensive cancer care, especially psychosocial care [12]. Hence, it is essential to contextualise patients' needs and experiences in LMICs to propose localised recommendations.

Advanced cancer patients have more unmet needs and challenges that differ from those with early-stage cancer due to the rapid disease progression [13]. Patients also reported having the highest symptom burden in the immediate postdiagnosis period and required efficient supportive care [14, 15]. Hence, this study aims to describe advanced cancer patients' unique experiences and needs in a multi-ethics, multi-religion upper-middle-income country during the first year postdiagnosis. By exploring patients' physical, psychological, social, and spiritual needs, this study aims to discuss potential gaps in the Malaysian health system and propose measures to improve cancer care.

## Methods

### Participant selection

Participants were recruited via the National Cancer Institute (NCI) and several cancer support groups from June 2021 to June 2022. NCI is a public institution and the main referral centre for cancer cases in Malaysia. Participants at NCI were recruited face-to-face at an outpatient clinic via convenience sampling, while recruitments from cancer support groups were based on a voluntary basis. Informed consent was obtained from all participants included in the study. All participants who consented completed the interview.

The inclusion criteria for participating in this study were as follows: (1) adults (≥ 18 years old); (2) diagnosed with stage 3 or 4 cancer within a year; (3) aware of own diagnosis and staging; (4) permanently residing in Malaysia. Participants with communication barriers were excluded from the study. Nineteen patients successfully participated in the study.

### Data collection

All participants took part in a semi-structured, in-depth interview guided by a topic guide. The topic guide was developed based on published literature and feedback from oncologists and palliative care specialists. The primary interviewer (ASJY) introduced herself as a PhD candidate and researcher and explained the purpose of the study. Then, she discussed and explored certain subjects in varying lengths and detail with the participants, including (1) physical, psychological, spiritual, and social needs and (2) experience of getting a diagnosis, receiving treatment, and living with advanced cancer. All interviews were conducted virtually either via telephone calls or video calls while patients were at home. Each interview lasted about 30 min–1 h. Family members or friends might be present during some of the interviews. No repeat interview was carried out. The interviews were audio-recorded and transcribed verbatim. These transcripts were also accompanied by field notes that the researcher took throughout the interview process.

This study has obtained ethical approval from the Ministry of Health Medical Research and Ethics Committee (NMRR-20-2404-56932) and Monash University Human Research Ethics Committee (Project ID: 28923).

### Analysis

This study adopted a critical realist paradigm and the results were analysed using iterative thematic approach. Line-by-line coding was applied to all the de-identified transcripts by the lead author (ASJY) using NVivo (released in July 2021) [16]. Insights from the field notes and memos were used to complement the coding process. The codes, data interpretations, and data saturations were discussed between the researchers (ASJY, MWLC). Data saturation was reached at the nineteenth participants and data collection was stopped. Constant comparisons between the transcripts were employed throughout the coding process to develop themes. The themes developed were subjected to a triangulation process to ensure the validity and rigour of the findings. The final findings were presented to all research team members (including public health researchers and palliative care specialist) for verification and endorsement. The study was reported according to the Consolidated Criteria for Reporting Qualitative Studies (COREQ) guideline [17].

### Background of researchers

ASJY (female) is a registered pharmacist and researcher with training in qualitative and quantitative research methods. MWLC (male) is a registered pharmacist with clinical experience in public hospitals and a public health researcher focussing on palliative care. EH (male) is a physician, researcher, and leader of a non-profit organisation which provides home-based palliative care. SLT (female) is a registered pharmacist and lecturer with a research interest in palliative care.

## Results

The mean age of the 19 participants was 53 years old (range 23–72) (Table 1). The majority of the participants were Malay (52.6%), from the bottom 40% of income strata (earning less than RM3000 per month) (52.6%), and without tertiary education (50%). Patients with different types of cancers were interviewed. Most of them were recruited from a public hospital (89.5%) and were within the first year after diagnosis (89.5%).

**Table 1:** Demographic characteristics of participants

Participants (*n* = 19)									
Age		Gender		Ethnicity		Religion		Education level^a^	
< 40	3	Male	10	Malay	10	Islam	12	No formal education	1
40–60	12	Female	9	Chinese	4	Buddhism	3	Primary level	4
> 60	4			Others	3	Hinduism	2	Secondary level	4
				Indian	2	Christianity	1	Tertiary level (University)	9
						Others	1		

Monthly household income^a^		Cancer types		Cancer staging		Time since diagnosis		Recruitment sites		
≤ RM999	4	Colorectal	6	Stage III	10	1–6 months	9	Hospital		17
RM1000- RM2999	6	Female reproductive	4	Stage IV	9	7–12 months	8	Support groups		2
RM3000- RM4999	1	Nasopharyngeal	3			> 1 year^b^	2			
> RM7000	6	Breast	2							
		Male reproductive	2							
		Lung	1							
		Lymphoma	1							

^a^Contains missing data

^b^Recruited to include their experiences with cancer support groups

Four themes were developed from the analysis of the coded data. These themes were “disruption to daily living”, “psychosocial and spiritual support system”, “information needs”, and “financial needs”.

### Theme 1: Disruption to daily living

While clinicians often emphasise clinical markers or symptoms, the impact on patients was demonstrated by the disruption to their daily lives. Patients who were newly diagnosed and had not started treatment reported that they were still functional and could carry out their everyday activities.

I mean, for the time being, I'm very mobile. The only thing is maybe during the treatment, there is side effect, that is the time maybe I need [help]. But with my alternative medicine, so far I, I don't really need anybody to... I mean, I'm I'm mobile. I can still drive, I can do my housework. (PT04, 70, retired radiographer)

However, after starting treatment, it affected patients the most when symptoms or treatment side effects profoundly restricted their daily functioning and abilities to fulfil responsibilities.

It [chemotherapy] destroys your whatever remaining days. So you cannot function, that is so sick. I think when I went through my chemotherapy, I was sick many of those days. So I don’t think I’ll go through chemotherapy [again]. (PT16, 65, retired engineer)

Treatment significantly impacted patients' lifestyles, especially after events such as major operations. One patient lamented how the use of a stoma bag impacted her work and leisure activities outside the house.

When it [faeces] comes out a lot, I had to go to the toilet and that's difficult. The toilet is normally siting or squad toilet, so it's harder. If I'm at home, I'll just use the sink. When I'm outside, at the workplace, I need to use the toilet, it's hard, even so with the attire at work. (PT08, 31, retail assistant)

Patients wished to feel normal and carry out their daily activities as before. One patient mentioned that he felt normal when his friends treated him indifferently and enjoyed their social activities together as they used to. Patients felt lonely when they were too weak to move around or do things they enjoyed.

And then this is you become lonely when you have this kind [of] diseases. You are not capable of moving, your mobility is limited. You feel lonely, very lonely. (PT10, 58)

Since women are culturally expected to be homemakers, cancer disproportionately affected younger women with children as they often felt the emotional burden when they had to neglect their household responsibilities of housekeeping and the care of children due to their physical conditions.

When you feel so sick, you feel that you neglected the whole family. You feel very bad… like that 12 days when I was so sick, I was on the bed all the time. […] I feel sad because I don't want my children to see me like that. But at the same time, I have no strength to… to show them that I'm okay. (PT18, 48, business owner)

Despite having a healthcare system that is universally accessible, accessing care still brought significant disruptions to daily living for some patients. Patients staying far from the hospitals or in different states had to get extra time off work and spend more money on transportation and even accommodation. It was helpful for such patients when the hospital allowed them to be warded despite undergoing outpatient treatment. Financial support was reported to alleviate the disruption to daily living. Adequate financial support would ease patients' burden as they did not need to worry about providing for their families. They could hire caretakers to relieve them from some of their household responsibilities, and they would have easier access to healthcare services with better facilities.

If you’re very rich, you can hire a helper. Or if your siblings are not working, they can take care of you. If you don’t have money, it’s useless. If you don’t have money, only your parents will take care of you. (PT09, 47, labourer)

### Theme 2: Psychosocial and spiritual support system

Other than addressing their clinical symptoms, patients need to be seen as a person by healthcare providers (HCPs). Some patients felt that the HCPs had been supportive and personal to them, while some felt that their doctors were merely managing their clinical symptoms and not other concerns.

You could have challenges emotionally, but it's not addressed in a normal clinical setting, then it's not addressed unless you come up with you... you you have... you show signs of psychosis. (PT01, 59, pharmacist)

Anxiety and depression were commonly reported among patients, and they looked different at different stages of their cancer journey. At the initial stage of diagnosis, patients were depressed as they struggled to accept the diagnosis and make tough decisions on the treatment. The feelings were not resolved even after initial remission as there was a constant fear of recurrence or metastasis.

So that that I'm a little bit anxious to find out the results in September because that would be one year after the removal. But obviously, if those results come out negative and show that it's spread somewhere, then it's really going to, I think, worry me more. (PT02, 60)

In the face of these emotions, family's and friends' care and assurance brought comfort to patients. Simple gestures such as calling to check on them, preparing food, and visiting them in hospitals made patients feel cared for and not alone in the journey.

All my children are supportive. Like one of my children has taken unpaid leave for 2 months… to look after me… until I’m strong. […] Although there are a lot of people at home, I have many other children, that’s the support that makes me moved. Even though I have told him no need, but he insisted, so I just follow… (PT05, 53, homemaker)

Religious patients found it easier to accept the diagnosis as they saw the sickness as either the will of God or a test from God.

Not everyone gets cancer, but I think I get tested because God loves me, so I just continue with my life, be positive. Maybe there is something that God wants to give to me one day, I don't know. (PT08, 31, retail assistant)

Another prominent trend that arose was the need for hope among the patients. Despite being diagnosed with stage 3 or 4 cancer, they still wanted to know that things would get better and that they could be free from cancer one day. Often, experiential sharing from family members or friends and even meeting other cancer patients gave hope and assurance to patients to continue treatment.

So I thought it’s good that I can talk to someone who has been through it, I can feel that his experience will be helpful to me. […] So such sharing lessens my worries regarding my treatment. (PT12, 58, machine operator)

Social activities had a positive impact on the psychological well-being of the patients. Cancer support groups empowered cancer patients via all the activities conducted. Some saw these activities as excellent distractions to help them worry less about their disease.

And I I join [cancer support group], as you know, to get to know... Actually it's to be distracted. […] There were a lot of activities in [cancer support group] that helped me to get through. […] you know, one of the activities for that that will go to Congress is to have a fashion show during the luncheon. So we practised like hell. (PT01, 59, pharmacist)

Patients also felt the need to do something to feel they were in control. Some did so by modifying their diet and exercising, while others resorted to complementary and alternative medicine (CAM).

And then now I'm given one month appointment. […] I have adenocarcinoma. What do you want me to do? Sit at home, doing nothing? […] I must do something, whether it work, it doesn't work, at least my mind is clear… I don't I don't just sit down and wait. (PT04, 70, retired radiographer)

While most patients managed to manoeuvre through their psychosocial needs via support from friends or family, some barriers were identified within the support system. Family support was hard to come by for socially isolated patients, and professional counselling services were not readily accessible. On the other side of the coin, patients often felt they were burdens to others when they had to depend on them for daily chores such as preparing their meals, bringing them around, and cleaning the house. They felt helpless because they could not lessen the burden of the caregivers, usually their loved ones.

But I feel sad sometimes. My children went to work, when she comes home, she has to cook. It’s not easy for her. I cannot do any work, I wanted to help a bit, they also won't let. They scolded. […] I don’t think of living long, just don’t want to trouble anyone. I don’t want to trouble my children. If I die, I die, don’t want to trouble. (PT06, 49, homemaker)

### Theme 3: Information needs

Patients needed information about their disease, end-of-life (EoL) diagnosis, managing symptoms, side effects, diet, and nursing care. This information was vital, so patients knew what to expect during the treatment, which gave them assurance.

Insufficient information regarding nursing care and symptom management can lead to anxiety and reduced patient QoL.

First time it was not detailed enough. But now I'm ok. [In which aspect is it not detailed?] how to use it [stoma bag], how to clean it, they didn’t mention, and they don’t say what I can't do, they just let me go home to figure out myself. (PT08, 31, retail assistant)

Patients typically prefer their doctors as the primary source of information, but this was limited by the consultation time.

And also not much advice from them, like what to eat, how to heal faster, you know? Mm-hmm. They just like telling you exercise la… and all that... they don't really tell you in depth. Mm-hmm. It's. Maybe maybe you know la, they got so many patients to see, they don’t have time to spend with you, maybe it’s that. (PT11, 67, retired admin executive)

Some patients felt they were not getting sufficient information from HCPs due to language barriers or low health literacy, whereby they were unsure what to ask. Hence, they turned to other sources such as family, friends, support groups or online resources for information. This increased the chances of misinformation if the patients did not have sufficient health literacy to process the information.

But some people, when you tell them what’s the [side] effect, they become afraid. Do you know… there are 2 or 3 patients that went through one time chemo and then they decided not to go through it, because of this thing, because when they start asking about the information online, they they start getting people telling them your your fingernails have become become black, and you're losing your hair, and you are having very… fatigue, you feel very tired. So they don't want to go through that because they don't want to lose their hair. (PT18, 48, business owner)

As much as cancer support groups can be valuable avenues to provide emotional and informational support, there is a weak linkage between the hospital and support groups. Most patients were not given information on support groups unless they took the initiative to find out.

I'm lucky because I got in touch with [a cancer society] and through them, I learned... I learned about [a cancer support group]. But if I were, if I didn't, if I didn't... say if I didn't have that, if I didn't find it out, I probably don't have access to a support group. (PT01, 59, pharmacist)

Issues such as family members withholding information from patients were also not uncommon. Some family members preferred the doctors to go through them before communicating with patients.

Initially they [the doctors] told me. Then when my children came, he told them. Initially I know la. Then my children were angry why the doctor told me, it’s sufficient if he told them first. Because they are scared I will be worried after hearing. (PT06, 49, homemaker)

When information regarding EoL diagnosis was withheld from patients, it affected their choices. For instance, some patients expressed their wish to avoid chemotherapy at their EoL due to the suffering it brought. However, if they were not aware of the EoL diagnosis, they might have agreed to undergo more aggressive treatments and compromised their QoL.

Effective communication is the key to building trust between patient and their HCPs. Some patients trusted their doctors fully and decided on their management plan wholly based on the doctor's advice.

We have to follow what the doctor says, because doctor knows better what's the outlook like. we don’t know because we don’t have knowledge regarding this disease, I only know I have cancer, but I don’t know how it's like inside, how it's like if later it spreads. (PT08, 31, retail assistant)

However, mistrust happened when patients were given advice that contradicted their beliefs. One such instance was when patients reported feeling judged or not being respected for their beliefs when they shared their use of CAM. Consequently, they dropped out of the health system to seek CAM and only returned later when their cancer deteriorated.

[Before you decided against surgery, did you tell your doctor about your alternative treatment?] Yeah, I told them I'm… but they... I know they will laugh off and say, “Why, why go and do that? It’s no use?” And then they will tell you that. (PT11, 67, retired admin executive)

### Theme 4: Financial needs

Patients have financial needs due to several reasons. Firstly, they had to bear the cost of treatment. Unfortunately, timely access to cancer care was sometimes dependent on the patient's financial ability. Due to the long waiting time and limited facilities in public hospitals, patients had to bear the high medical cost in the private sector to prevent delays in diagnosis or treatment.

I went to [public hospital] actually, since I want to do CT scan, I have to wait for several days, half a month because [the public hospital] always packed. So they just advised me to do CT scan at private [hospital]. […] And appointment dates would be full most of the time. Let's say if I want to take a CT scan, the CT scan would be minimum four to… minimum I think six months for me to take the CT scan. (PT13, 23, student)

If really someone want to save me, of course I will go to private hospital. But now I have no money and no one wants to help me, how can I go to private hospital. Private hospital you just go in for something simple, it will cost you more than RM1000, the cheapest also RM600. (PT09, 47, labourer)

Some patients with private medical insurance subscriptions were forced to transition from private to public facilities when they exceeded the limits of their medical insurance or because the services they needed were not being covered under the insurance, such as genetic testing and targeted therapy.

Even though the government highly subsidises the medical cost in the public sector, there are also some services that patients have to pay out-of-pocket, such as breast reconstruction surgery and specific targeted therapies that often cost an exuberant amount of money.

The targeted medication is quite expensive, like Herceptin costs about RM47,000 and surgery maybe costs about… not too sure, but maybe about 10–15k. I’m not too sure. Yeah. The that is considered affordable compared to a private hospital or any other hospital. (PT18, 48, business owner)

Secondly, cancer affected the livelihood of the patients as they could no longer work when they were undergoing treatment. For permanent employees, patients could usually get medical leaves without pay cuts, but the impact was significant for those daily wage earners. The loss of income affects not only the patient but their family due to the opportunity cost of caregiving.

But my husband didn’t take leave for too long, like when I had my chemo cycles 1–3, he took leave for one week. For cycles 4 to 6, he had to take 2 weeks of leave. Every time after chemo, I couldn’t get up, he had to take leave for 2 weeks. So it affected the finance of the family, because my husband couldn’t go to work. (PT15, 43, homemaker)

Thirdly, the cost of living was increased due to the need to pay for additional transportation to the hospital, special dietary needs, nursing care and CAM. This was a twofold blow for patients as they struggled with the increased living cost while losing their ability to work.

I had to spend RM1500 per month on milk powder. That’s where I spent all my savings. Now it’s been 9 months I drink milk. Till now, I still can't eat solid food. (PT12, 58, machine operator)

The Covid-19 pandemic further impeded access to care in the public sector. Treatments were delayed as public hospitals had to prioritise Covid-19 patients. Patients with pressing symptoms were forced to bear a substantial out-of-pocket cost to receive treatment in private hospitals.

Because that time Covid cases were rising, thousands of cases daily, so they don’t do operation. They suggested me to have the operation at private [hospital] if I can afford. But if I can’t afford it, I have to wait until Covid cases reduce. But I did my operation at [private hospital]. Because its already so big, too big. (PT05, 53, homemaker)

To fulfil these financial needs, patients had to use their personal savings (including pension funds), borrow money from their family members or apply for financial aid. Patients reported getting financial aid from PERKESO, Zakat, MySalam, Jabatan Kebajikan and some NGOs such as MAKNA. Some patients expressed difficulties navigating the complex system to get financial aid because different financial aids had different requirements, and often, this information was not readily available.

Now I’m trying [a financial aid], but until now no news from them, I don't know why. they say they're busy because now got flood, their staff are… they are busy with flood project. They say ask me to hold on until… actually they [are supposed to reply within] 30 days but now they told me, I have to wait another one more month. Have to wait la. I have to look up lo, see any other financial aid. (PT11, 67, retired admin executive)

## Discussion

This study explored advanced cancer patients' lived experiences and needs within the first year postdiagnosis. Patients had a wide variety of needs throughout this time as they learned to cope and accept their diagnosis, decided on their treatment, accessed the health system to receive the care they needed, and lived with the impacts of their disease. These experiences and needs converged into four major themes: disruption to daily lives, psychosocial and spiritual support system, information needs, and financial needs.

The themes identified were consistent with a quantitative study conducted among breast cancer patients in Malaysia, which reported that they had the highest unmet supportive care needs in the psychological domain, followed by physical, patient care needs, and health information [18]. Studies have found that unmet needs in the early postdiagnosis stage of cancer negatively impact a patient's QoL [7, 19]. Hence, it is crucial to recognise these needs and provide sufficient supportive care right from the beginning of diagnosis. Greater supportive care is associated with better clinical outcomes [20] and improved QoL [21].

Clinicians should be aware of how patients experience cancer in their daily lives. Getting treatment is just one small aspect of the cancer experience for patients. They need other forms of support, such as counselling support, nursing care, fertility preservation, occupational therapy, and palliative care to minimise the impact of cancer on their daily living. For instance, rehabilitation or occupational therapy is essential to help them achieve maximum levels of independence. This improves patients' QoL because they often "felt like a burden" when they lost their physical independence or could not fulfil their family and societal roles [22]. Services such as home-based nursing care, hospice care, and respite services would fulfil patients' supportive care needs and, at the same time, relieve caregivers’ burden greatly [23–25]. However, several structural barriers exist to accessing these services in Malaysia. Previous studies have found that clinicians act as gatekeepers or referral points to other allied health services [26, 27]. Another barrier to accessing allied health services is the long-standing shortage of allied health professionals in the public sector, especially in the primary care setting. To illustrate the extent of the shortage, Malaysia only had 0.21 psychologists per 100,000 population in 2015 [28], which falls way behind the 1:5000 recommended ratio [29]. In view of the many non-medical needs of advanced cancer patients, clinicians should be proactive in assessing such needs and the health system should allow easy access to supportive care and other allied health services.

Cancer patients’ need for a myriad of information is consistent with findings from other systematic reviews, which showed that information need is one of the most prevalent unmet needs among patients [9] and their caregivers [10]. Clinicians and other allied HCPs are the preferred source of information [30, 31], but patients often reported receiving “generic advice” from them [32]. For instance, when told that there are no specific dietary restrictions during cancer treatment, patients frequently felt that the doctors were not providing sufficient information or addressing their concerns seriously, which could lead to mistrust and dissatisfaction. While the information is accurate, HCPs need to better understand patients' cultural and social expectations to ensure effective communication. In this instance, most ethnicities in Malaysia have cultural beliefs on food prohibited during cancer [33, 34] or other life events such as pregnancy or postpartum [35–37]. With this understanding, HCPs can spend some time debunking the "myths" that patients might have and tailor their advice according to cultural expectations while providing health information.

Similarly, there are cultural and traditional beliefs related to CAM use among the multi-ethnic Malaysian population [38]. Cancer patients use a wide variety of CAM, such as dietary supplementation, herbal products, spiritual healing, traditional Chinese medicine, and traditional Malay therapy [39–41]. This study revealed some of the rationales behind patients’ use of CAM, such as to get hope, to feel in control, to avoid aggressive treatment or merely due to misinformation. However, most patients did not disclose their use of CAM to their HCPs. This could lead to delays in presentation and diagnosis, non-adherence to treatment, and even dropping out from the health system [42]. Trust between clinicians and patients is pertinent to encouraging them to be open about their practice and concerns [43, 44]. Discussions with patients regarding the use of CAM require cultural understanding and "experience in listening, encouraging hope, and ability to convey empathy and compassion” [45].

This study provides insights into the financial concerns of cancer patients in Malaysia. In fact, financial toxicity caused by cancer has been well-established in the literature, particularly in LMICs and countries without universal health coverage [46, 47]. Malaysia has a unique dichotomous healthcare system consisting of the public and private sectors. The government highly subsidises medical fees in the public sector via a tax-funding mechanism, while the payment is via an out-of-pocket fee-for-service mechanism in the private sector. Even though most of the participants in this study were recruited from public hospitals where the medical fee is nominal, it has been exemplified how cancer still affected their living and livelihood. This is consistent with a previous quantitative study which reported that 33% of those who received treatment in public hospitals had to spend more than 30% of their annual household income one year after their cancer diagnosis [48]. Like the findings in this study, the majority of the expenditure (86%) was on CAM and other non-medical expenses such as transportation, meals, parking fees, and lodging. Hence, subsidising medical fees alone is insufficient as there are other indirect costs that affect patients' access to care. One possible way to mitigate the financial impact of cancer is to cover those from the lower-income group with some form of income replacement protection scheme because they are at a higher risk of experiencing financial toxicity [49–52]. For example, in Malaysia, MySalam scheme was introduced in 2019 as a national social health protection scheme that provides low-income beneficiaries with financial aid for critical illness diagnoses and hospital admissions [53]. This is definitely one of the ways forward. However, low public awareness [54], poor implementation [55], and the lack of governance [56] need to be addressed before the benefit of social health protection can be reaped. To conclude, it is crucial to recognise the financial factors that impede access to health services among the lower-income group and devise a comprehensive social health protection scheme.

Another observation was that many patients were transferred from the perceived higher-quality private sector to the lower-quality public sector when they could no longer afford the medical fee. This included patients who had subscriptions to private insurance schemes but had limited coverage or insufficient reimbursement policies [57]. Patients in this situation faced a double-whammy of sorts: first, they exhausted their financial resources, and then they got sent back to the public health system that they were trying to avoid in the first place. This offloading of patients also overwhelms the public sector, which has already been notorious for the long waiting time and the lack of resources [58]. So, to ensure universal health coverage with consistent quality, the public health system needs a sustainable health financing system, such as the much-advocated national health insurance scheme [59]. Under the insurance scheme, the private sector could be contracted to provide services that would later be reimbursed. This gives the Ministry of Health more control over the cost of treatment and coverage of the medical insurance scheme. Such a public–private partnership would also decongest the overburdened public sector as it allows the mobilisation and sharing of facilities and human resources from the private sector [60]. In short, the Malaysian health system needs an improved model of healthcare financing and a better integration of public and private health services.

### Strengths and limitations

The strength of this study lies in the extensive exploration of context-specific needs and barriers that patients experience in accessing cancer care. These barriers were discussed with the Malaysian health system in mind to propose corresponding recommendations for system improvement. This study also involved a diverse sample of interviewees regarding age, ethnicity, religion, income, education levels, and cancer types. This allows in-depth exploration of their experiences and needs, especially during the first year postdiagnosis. However, having said so, the findings might not be representative of the whole cancer population as different patients might have their own unique needs across the disease trajectory. Since the patients in this study were recruited from a single public hospital, their experiences and needs might vary from patients from other regions, private hospitals or patients who were not receiving treatment from the mainstream health system. Due to practical reasons, this study could not include certain groups of patients, such as those severely ill, cognitively impaired, and those with language or communication barriers. Besides that, interview transcripts were not returned to participants for checking, but the final findings were presented to all research members. Finally, telephone interviews might be inferior to face-to-face interviews due to the lack of rapport and limited interpretation of non-verbal data. Even though the interviews were not in-person, relationships were built prior. All the participants were recruited as part of a larger qualitative study (under review), where the same interviewer (AYSJ) administered a face-to-face survey for 30 min to one hour. On top of that, previous studies reported that telephone interviews do not produce lower-quality data than face-to-face [61, 62]. Moreover, it has added advantage of increased privacy for interviewees, which allows them to be more open to sharing sensitive topics, in this case, their emotional states [61, 63].

## Conclusion

Advanced cancer patients have many needs beyond clinical indicators, particularly in the areas of daily functioning, psychosocial, information, and finance. To fulfil these needs, stakeholders such as clinicians, allied HCP, social workers, cancer support groups, family members, and caregivers must first be aware of these needs and then join forces towards fulfilling these needs. In terms of the health system for cancer care, there is an imperative need to enhance access to supportive care, such as nursing care, palliative care, rehabilitation, and counselling support. HCPs should be more aware and sensitive to cultural contexts while providing health information to encourage shared decision-making with patients. The health system should also be proactive in assessing patients’ financial needs regarding income replacement protection schemes and other non-medical costs to ensure equity in accessing care.
